# The GLP-1 receptor agonist exenatide improves recovery from spinal cord injury by inducing macrophage polarization toward the M2 phenotype

**DOI:** 10.3389/fnins.2024.1342944

**Published:** 2024-02-15

**Authors:** Toshihiro Noguchi, Hiroyuki Katoh, Satoshi Nomura, Keiko Okada, Masahiko Watanabe

**Affiliations:** Department of Orthopaedic Surgery, Surgical Science, Tokai University School of Medicine, Isehara, Japan

**Keywords:** spinal cord injury, GLP-1 receptor agonist, exenatide, macrophage, inflammation

## Abstract

Although a wide variety of mechanisms take part in the secondary injury phase of spinal cord injury (SCI), inflammation is the most important factor implicated in the sequelae after SCI. Being central to the inflammation reaction, macrophages and their polarization are a topic that has garnered wide interest in the studies of SCI secondary injury. The glucagon-like peptide 1 (GLP-1) receptor agonist exenatide has been shown to enhance the endoplasmic reticulum stress response and improve motor function recovery after spinal cord injury (SCI). Since exenatide has also been reported to induce the production of M2 cells in models of cerebral infarction and neurodegenerative diseases, this study was conducted to examine the effects of exenatide administration on the inflammation process that ensues after spinal cord injury. In a rat contusion model of spinal cord injury, the exenatide group received a subcutaneous injection of 10 μg exenatide immediately after injury while those in the control group received 1 mL of phosphate-buffered saline. Quantitative RT-PCR and immunohistochemical staining were used to evaluate the effects of exenatide administration on the macrophages infiltrating the injured spinal cord, especially with regard to macrophage M1 and M2 profiles. The changes in hind limb motor function were assessed based on Basso, Beattie, Bresnahan locomotor rating scale (BBB scale) scores. The improvement in BBB scale scores was significantly higher in the exenatide group from day 7 after injury and onwards. Quantitative RT-PCR revealed an increase in the expression of M2 markers and anti-inflammatory interleukins in the exenatide group that was accompanied by a decrease in the expression of M1 markers and inflammatory cytokines. Immunohistochemical staining showed no significant difference in M1 macrophage numbers between the two groups, but a significantly higher number of M2 macrophages was observed in the exenatide group on day 3 after injury. Our findings suggest that exenatide administration promoted the number of M2-phenotype macrophages after SCI, which may have led to the observed improvement in hind limb motor function in a rat model of SCI.

## 1 Introduction

Traumatic spinal cord injury (SCI) can be broadly subdivided into two phases: the primary injury directly caused by a traumatic external force, and the secondary injury that involves a complex vascular, cellular, and biochemical cascade that leads to necrosis and apoptosis of surrounding cells affecting a wider area (Beattie et al., 2000; Oyinbo, 2011; Borgens and Liu-Snyder, 2012). As the primary injury is unavoidable, treatment for SCI mainly focuses on mitigating the secondary injury. This study aims to establish a treatment for SCI using a drug that is already approved for use in the Japanese market for the treatment of type-2 diabetes. Glucagon-like peptide 1 (GLP-1) reduces blood sugar levels by stimulating insulin secretion from pancreatic islet β cells, and GLP-1 receptor agonists function by binding to GLP-1 receptors distributed throughout the body, including the central nervous system.

Various studies have examined the effects of multiple GLP-1 receptor agonists for the treatment of several neurological conditions and have reported beneficial effects (Graaf et al., 2016; Zhao et al., 2021; Kopp et al., 2022). While GLP-1 receptor agonists have been approved as a treatment for type-2 diabetes, recent research has uncovered that its effects are wide-ranging. In type-2 diabetes, pancreatic islet cell function declines due to chronic nonspecific inflammatory reactions including oxidative stress, endoplasmic reticulum stress, and mitochondrial dysfunction (Böni-Schnetzler and Meier, 2019), and the anti-inflammatory properties of GLP-1 receptor agonists improve islet cell function (Nauck et al., 2021). This anti-inflammatory function has benefits in the central nervous system as well (Diz-Chaves et al., 2022), and GLP-1 receptor agonists have been shown to be neuroprotective in animal models of Alzheimer’s disease (Perry and Greig, 2002; Reich and Holscher, 2022; Holscher, 2022a; Perry et al., 2023), Parkinson’s disease (Aviles-Olmos et al., 2013; Feng et al., 2018; Reich and Holscher, 2022; Holscher, 2022b), epilepsy (Wang et al., 2018), and stroke (Li et al., 2009; Teramoto et al., 2011). The robust data gained from animal studies has prompted the examination of GLP-1 receptor agonists in clinical trials of Alzheimer’s disease (Gerstein et al., 2019; Cukierman-Yaffe et al., 2020) and Parkinson’s disease (Aviles-Olmos et al., 2013, 2014) with promising results.

The neurotrophic/neuroprotective activities of GLP-1 receptor agonists have also been reported in the field of traumatic brain injury (Combs, 2015; Li Y et al., 2015; Glotfelty et al., 2019). This is highly relevant because the neuroinflammatory cascade seen in the secondary phase of traumatic brain injury mirrors that of SCI. In the secondary phase of injury in both the brain and the spinal cord which begins immediately following the primary injury, there are multiple factors that come into play, including vascular damage of the blood–brain/spinal cord barrier that causes edema, accumulation of excitotoxic neurotransmitters and free radicals that exacerbate inflammation, calcium influx as well as lipid peroxidation that collectively cause wide-spread necrotic cell death (Popovich et al., 2002; Donnelly and Popovich, 2008; Oyinbo, 2011; Chavez-Gala et al., 2015; von Leden et al., 2017). In the subacute to chronic phase of secondary injury, apoptosis, demyelination of surviving axons, axonal dieback, and Wallerian degeneration lead to loss of tissue and matrix remodeling that creates a cavity surrounded by a glial scar (Katoh et al., 2019). While stem cell therapy is a hot topic in the field of SCI research, many more patients could potentially benefit if the devastating effects of secondary injury could be mitigated; the use of GLP-1 receptor agonists is one of drugs being looked into as a potential pharmacological agent to protect against secondary injury.

While there is a diversity of GLP-1 receptor agonists, exenatide is the drug most studied, possibly due to its relatively high penetration of the blood–brain/spinal cord barrier (Athauda et al., 2017; Salameh et al., 2020). We previously reported that exenatide enhanced the endoplasmic reticulum (ER) stress response and led to the recovery of motor function after SCI in a rat model of spinal cord contusion injury (Nomura et al., 2023). While there are many SCI animal models such as precision cut injuries (transections), compression injuries (aneurysm clips), and contusion injuries (weight drop injuries), we have selected contusion injuries because they are considered to be reproducible and faithful models of human SCI that are caused by falls or physical impacts (Kjell and Olson, 2016). This study was conducted similarly to our previous study, but by a different primary researcher in a different set of animals, with the aim to confirm the benefits of exenatide and to examine its effect on the inflammatory response after SCI, especially on the polarity of infiltrating macrophages.

In the SCI secondary injury process, monocytes derived from the blood and bone marrow differentiate into macrophages and are recruited at the injury site and join the microglia endogenous to the spinal cord. Two macrophage phenotypes have been reported: “classically” activated M1 cells involved in inflammation and tissue damage, and “alternatively” activated M2 cells that reduce inflammation and promote tissue repair (Kigerl et al., 2009; Chavez-Gala et al., 2015; Gensel and Zhang, 2015). The involvement of exenatide in macrophage polarization has been noted in several studies (Shiraishi et al., 2012; Darsalia et al., 2014; Buldak et al., 2016; Wu et al., 2017), and here we report on the effects of exenatide on macrophage polarization in the injured spinal cord.

## 2 Materials and methods

### 2.1 Rat model of SCI

All animal experiments were conducted in accordance with the National Institutes of Health Guidelines for the Care and Use of Laboratory Animals and were approved by the Animal Experimentation Committee of Tokai University School of Medicine (approval number: 191010). Female Sprague–Dawley rats (10-week-old, weight: 280–320 g) procured from CLEA Japan, Inc., (Kanagawa, Japan) were placed under general anesthesia by inhalation of 4% isoflurane. The dorsal area of each animal was shaved, and an incision was made in the skin under aseptic conditions. The subcutaneous fat and paraspinal muscles were separated from the midline to expose the lower thoracic spine and a laminectomy of the 10th thoracic vertebra was performed to expose the dura mater. A contusion SCI was created using a spinal cord impactor (Infinite Horizon Impactor, Precision Systems & Instrumentation Lexington, KY, United States) with a force of 200 Kdyne. The paraspinal muscles and skin were then sutured and the animal was awakened from anesthesia.

Immediately after SCI, animals in the exenatide group were subcutaneously administered 10 μg of exenatide while those in the control group received 1 mL of phosphate-buffered saline (PBS) only. In our previous study, 10 μg of exenatide was administered immediately after injury and on day seven. Considering that the main effects of exenatide in our previous study were observed in the first few days, and also considering that macrophage infiltration into the spinal cord begins immediately after SCI, we decided to administer exenatide only once immediately after injury. The exenatide used in this study is Byetta® (purchased from AstraZeneca Pharmaceuticals LP, Cambridge, United States), which is a regulatory agency-approved exenatide for the treatment of type 2 diabetes to be administered at a dosage of 5 ug subcutaneously twice daily that can be elevated to 10 ug subcutaneously twice daily after a month. In the current rat study, the dosage of 10 ug was derived from past studies (Teramoto et al., 2011; Li H et al., 2015). Notably, this rat dose is considerably higher than the routine human dose, based on interspecies allometric scaling based on body surface area for dose conversion from animal to human studies in line with US FDA Guidelines (U.S. Department of Health and Human Services Food and Drug Administration Center for Drug Evaluation and Research (CDER), 2005). Although an equivalent dose to the one used in our rats could not be safely administered to humans, our selected rat dose provides a starting point for the evaluation of the efficacy of exenatide in SCI.

To address the dysuria that develops after SCI, each animal was given a bladder massage twice daily until voluntary urination was confirmed. Animals that developed cystitis or wound infection were excluded from the study. All animals were handled and housed according to a protocol approved by the Tokai University School of Medicine Life Science Support Center.

### 2.2 Sample collection

On days 1, 3, 7, and 14 after injury, animals were euthanized by inducing deep anesthesia using 4% isoflurane and then intracardially perfused with PBS before harvesting the spinal cords. Samples for histological investigation were perfused and fixed with 2% paraformaldehyde (PFA) and then dehydrated in a stepwise process that involved incubating at 4°C in 4% PFA, 10% sucrose solution, 15% sucrose solution, and 20% sucrose solution for 24 h each.

### 2.3 Quantitative RT-PCR

For quantitative reverse transcription polymerase chain reaction (RT-PCR), a 6 mm section centered on the injury epicenter was resected from the injured spinal cord. RNA was extracted and purified using the RNeasy Mini Kit (Qiagen, Japan), and the High-Capacity cDNA Reverse Transcription Kit (Applied Biosystems™ Thermo Fisher Scientific, Japan) was used to reverse-transcribe single-stranded cDNA from the isolated mRNA. Real-time PCR assays were performed on a LightCycler^®^ 480 system (Roche Diagnostics, Japan) using the Fast SYBR™ Green Master Mix (Applied Biosystems™ Thermo Fisher Scientific, Japan).

Inducible nitric oxide synthase (iNOS), CD16, and CD86 mRNA levels were assessed as M1 macrophage markers and Arginase 1, CD163, and CD206 mRNA levels were assessed as M2 macrophage markers. We also assayed for tumor necrosis factor α (TNFα), interleukin (IL)-1β, IL-4, and IL-10 mRNA levels. β-actin was used as the internal control. For each sample, the mean values of the assay measurements from two wells (*n* = 5) were used. The following primers designed for *Rattus norvegicus* genome were:

**Table 1:** 

iNOS	Forward; GCCCAGAGTCTCTAGACCTCAA
	Reverse; CATGGTGAACACGTTCTTGG
CD16	Forward; CAGCTAGACGTCCATGCAGA
	Reverse; TGGCATCTCAGACGAATGG
CD86	Forward; AGTGTTTGAAGATGCAGAACCA
	Reverse; CTGTCCTGCTTGGACTCACA
Arginase1	Forward; CCGCAGCATTAAGGAAAGC
	Reverse; CCCGTGGTCTCTCACATTG
CD163	Forward; ATGGGGAAGGCACAACTG
	Reverse; TCAGATCCGCTCCGTCTAA
CD206	Forward; AACAACCAGGCAGGAGGACTG
	Reverse; CAGTGGTTGCTCACAAGCTC
TNF-α	Forward; CGTAGCCCACGTCGTAGC
	Reverse; GGTTGTCTTTGAGATCCATGC
IL-1β	Forward; AGCTTCAGGAAGGCAGTGTC
	Reverse; TCCCACGAGTCACAGAGGA
IL-4	Forward; TCCTTACGGCAACAAGGAAC
	Reverse; TCTTCAAGCACGGAGGTACA
IL-10	Forward; CAGATTCCTTACTGCAGGACTTTA
	Reverse; CAAATGCTCCTTGATTTCTGG
βactin	Forward; TAAAACGCAGCTCAGTAACA
	Reverse; ATTGCTGACAGGATGCAGAA

### 2.4 Immunohistochemical staining

The spinal cords were embedded in Optimal Cutting Temperature compound (Sakura Finetek, Japan), frozen using liquid nitrogen, and sectioned on a cryostat microtome at a thickness of 10 μm. Considering the width of the tip of the IH Impactor (2 mm) as the width of the injury epicenter, spinal cord sample sections were prepared within 5 mm of the epicenter on the caudal side.

Sections were washed thrice in PBS for 10 min, blocked with 5% normal goat serum in PBS at 24°C for 60 min, and then washed again for 10 min before applying anti-macrophage marker antibodies (mouse anti-Iba1, Abcam, 1:100; rabbit anti-iNOS, Abcam, 1:200; goat anti-Arginase 1, Abcam, 1:200) and incubating the sections overnight at 4°C.

The following day, the sections were washed with PBS, the complementary fluorescent secondary antibodies were applied (Iba1: Alexa Fluor 594-conjugated, anti-mouse, 1:800; iNOS: Alexa Fluor 488-conjugated, anti-rabbit, 1:800; Arginase 1: Alexa Fluor 488-conjugated, anti-goat, 1:800), and the sections were incubated in a dark room at 24°C for 60 min. Nuclear staining and mounting were performed using VECTASHIELD Antifade Mounting Medium with DAPI (Vector Laboratories). Stained sections were viewed using a confocal laser microscope (LSM 700, Carl Zeiss) and analyzed using the ZEN 2009 software (Carl Zeiss). The dorsal funiculus was examined in five consecutive sections, and the total number of Iba1+ iNOS+ M1 macrophages and Iba1+ Arginase 1+ M2 macrophages were manually quantified. For each animal, the mean values of measurements by three examiners (*n* = 5) were used.

### 2.5 Behavioral analysis

Hind limb motor function was assessed based on the Basso, Beattie, Bresnahan locomotor rating scale (BBB scale) (Basso et al., 1995) scores on days 1, 3, 5, 7, 9, 11, 13, and 14 after injury (*n* = 5). Mean values of measurements by three examiners (*n* = 5) were used. Locomotion was observed for 5 min while rats ambulated freely in an open field.

### 2.6 Statistical analysis

Results are presented as the mean ± standard deviation values, and sample groups were compared using the Mann–Whitney *U*-test. IBM SPSS Statistics for Windows, version 23.0 (IBM Corp., Armonk, NY, United States) was used for all statistical analyses, and *p* < 0.05 was considered indicative of a statistically significant difference.

## 3 Results

**Figure 1:**
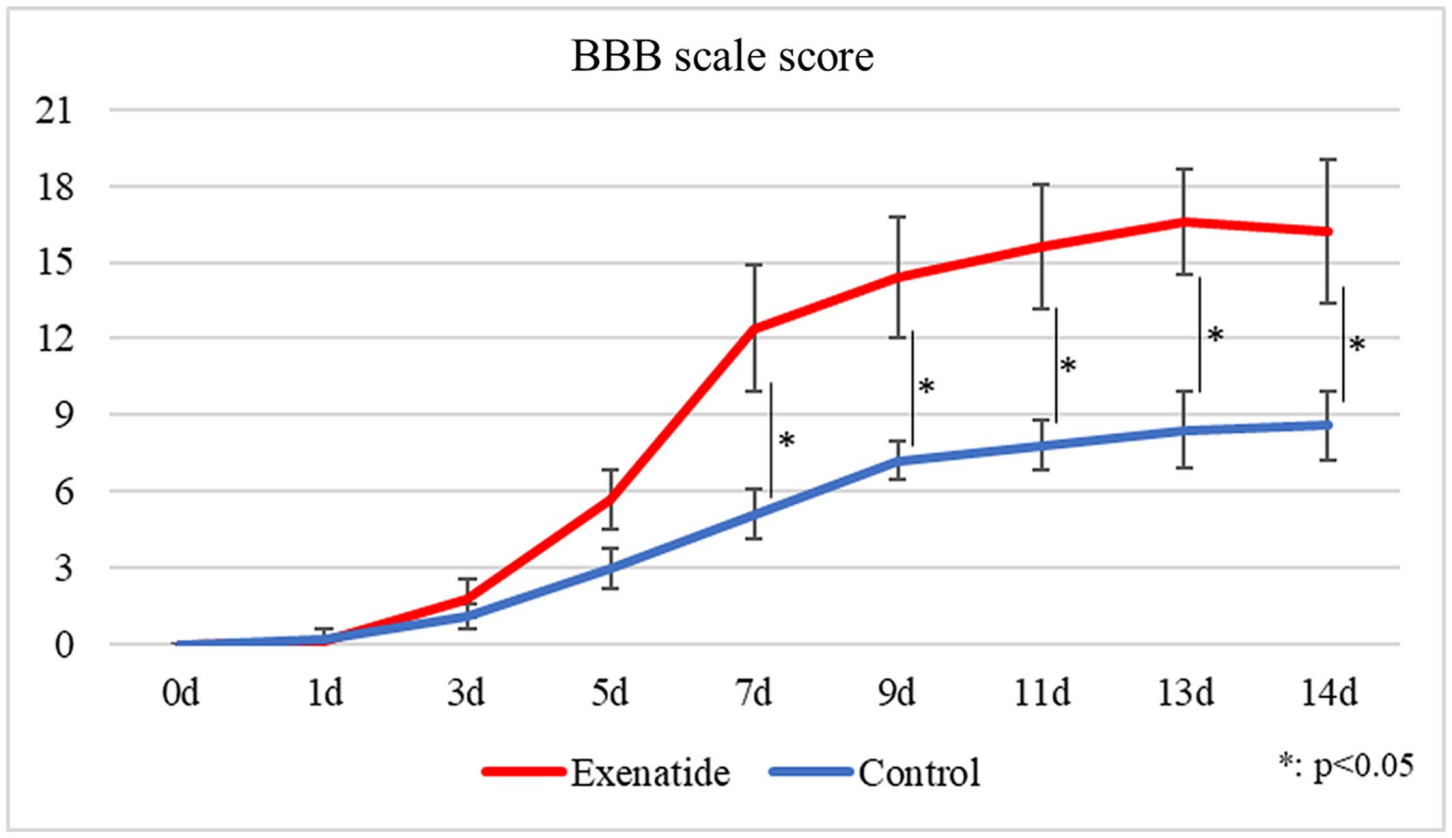
Change in BBB scale scores over time. BBB scale scores improved over time in both the exenatide and control groups, but there was significantly greater improvement in the exenatide group from day 7 after injury onwards. *n* = 5; error bars: SDs; ^*^p < 0.05, ^**^p < 0.01.

The BBB motor score evaluates hindlimb motor function on a scale from 0 to 21, with higher scores indicating higher function, i.e., improved hindlimb movement. While the BBB scale scores of both the exenatide and the control groups started from 0 immediately after injury and gradually improved over time, there was significantly greater improvement in the exenatide group compared to the control group from day 7 after injury and onwards (*p* < 0.05; Figure 1).

**Figure 2:**
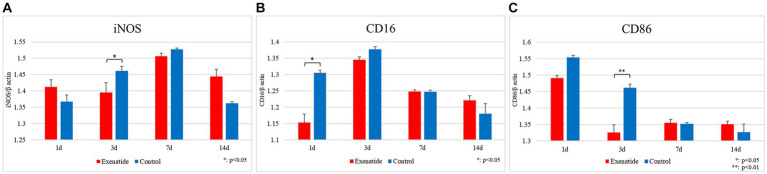
mRNA expression levels of M1 markers in the injured spinal cord. Compared to the control group, the exenatide group showed significantly lower iNOS mRNA levels on day 3 after injury **(A)**, lower CD16 levels on day 1 after injury **(B)**, and lower CD86 levels on day 3 after injury **(C)**. β-actin was used as the control. *n* = 5; error bars: SDs; ^*^p < 0.05, ^**^p < 0.01.

**Figure 3:**
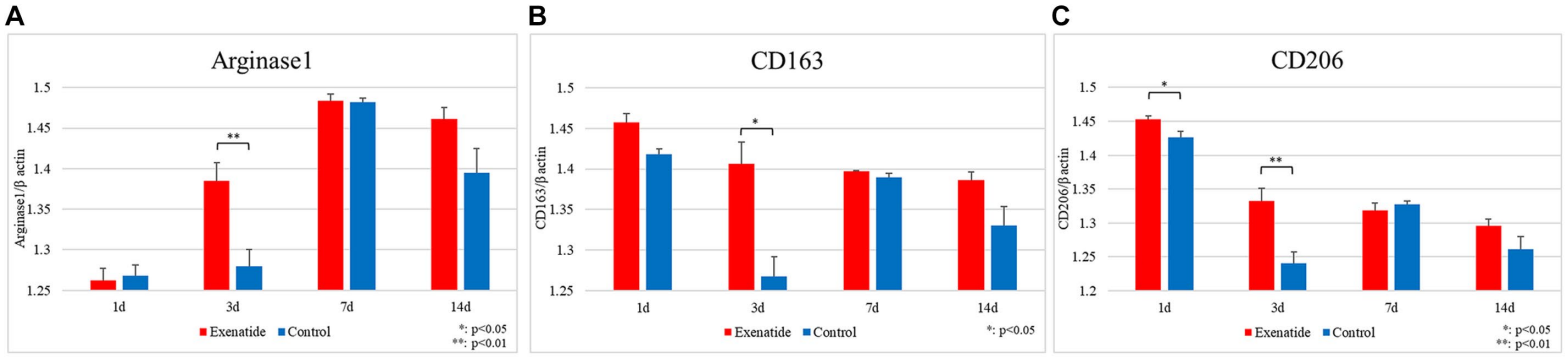
mRNA expression levels of M2 markers in the injured spinal cord. Compared to the control group, the exenatide group showed significantly higher Arginase 1 mRNA levels **(A)** and higher CD163 levels **(B)** on day 3 after injury, and significantly higher CD206 levels on days 1 and 3 after injury **(C)**. β-actin was used as the control. *n* = 5; error bars: SDs; ^*^p < 0.05, ^**^p < 0.01.

RT-PCR of the spinal cord tissue was conducted in order to analyze changes in the characteristics of macrophage infiltrating the injured spinal cord. The pro-inflammatory M1 macrophage markers demonstrated significant reductions in the exenatide group compared to the control group, with the exenatide group showing significantly lower iNOS mRNA levels on day 3 after injury (*p* < 0.05; Figure 2A), significantly lower CD16 mRNA levels on day 1 after injury (*p* < 0.05; Figure 2B), and significantly lower CD86 mRNA levels on day 3 after injury (*p* < 0.01; Figure 2C). On the other hand, the anti-inflammatory M2 macrophage markers demonstrated significant increases in the exenatide group compared to the control group, with the exenatide group showing significantly higher Arginase 1 and CD163 mRNA levels on day 3 after injury (*p* < 0.01, Figure 3A and *p* < 0.05, Figure 3B, respectively), and significantly higher CD206 mRNA levels on days 1 and 3 after injury (*p* < 0.05 and *p* < 0.01, respectively) (Figure 3C).

**Figure 4:**
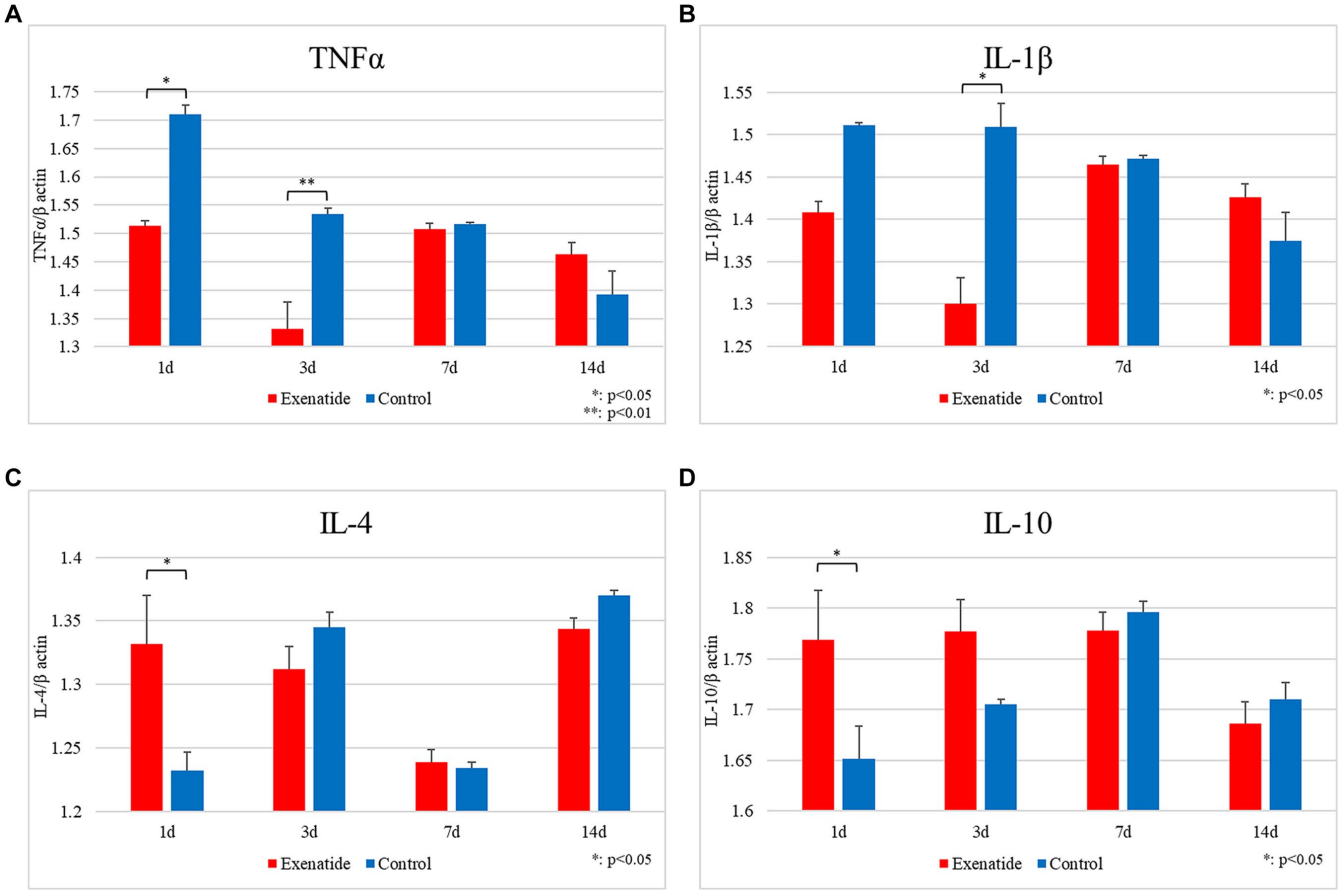
mRNA expression levels of various cytokines in SCI. Compared to the control group, the mRNA expression levels of pro-inflammatory cytokines were lower in the exenatide group, with TNFα significantly lower on days 1 and 3 after injury **(A)** and IL-1β significantly lower on day 1 after injury **(B)**. Conversely, the mRNA expression levels of anti-inflammatory cytokines were higher in the exenatide group, with both IL-4 **(C)** and IL-10 **(D)** significantly higher on day 1 after injury. β-actin was used as the control; *n* = 5; error bars: SDs; ^*^p < 0.05, ^**^p < 0.01.

In order to examine the effects that the change in macrophage profile had upon the inflammatory process within the injured spinal cord, RT-PCR of inflammatory markers were also performed. The mRNA levels of the pro-inflammatory markers were significantly lower in the exenatide group compared to the control group, with TNFα mRNA levels significantly lower on days 1 and 3 after injury (*p* < 0.05 and *p* < 0.01, respectively; Figure 4A), and IL-1β levels lower on day 3 after injury (*p* < 0.05; Figure 4B). Conversely, the mRNA levels of the anti-inflammatory markers IL-4 and IL-10 were significantly higher in the exenatide group compared to the control group on day 1 after injury (*p* < 0.05; Figures 4C,D).

**Figure 5:**
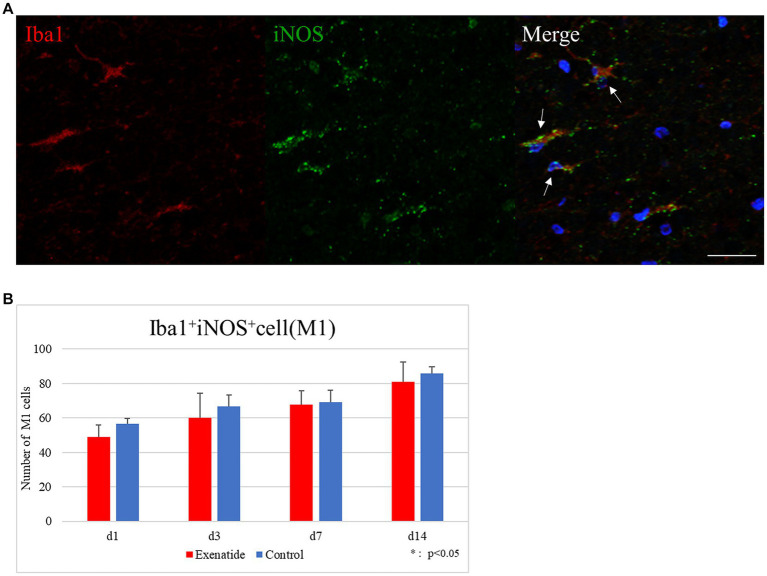
M1 macrophages in the injured spinal cord. **(A)** Representative immunohistochemical staining results for the M1 profile. DAPI: blue, Iba1: red, iNOS: green. Scale bar: 10 μm. **(B)** The number of Iba1+ iNOS+ cells in the posterior funiculus showing no difference among exenatide and control groups. *n* = 5; error bar: SD.

**Figure 6:**
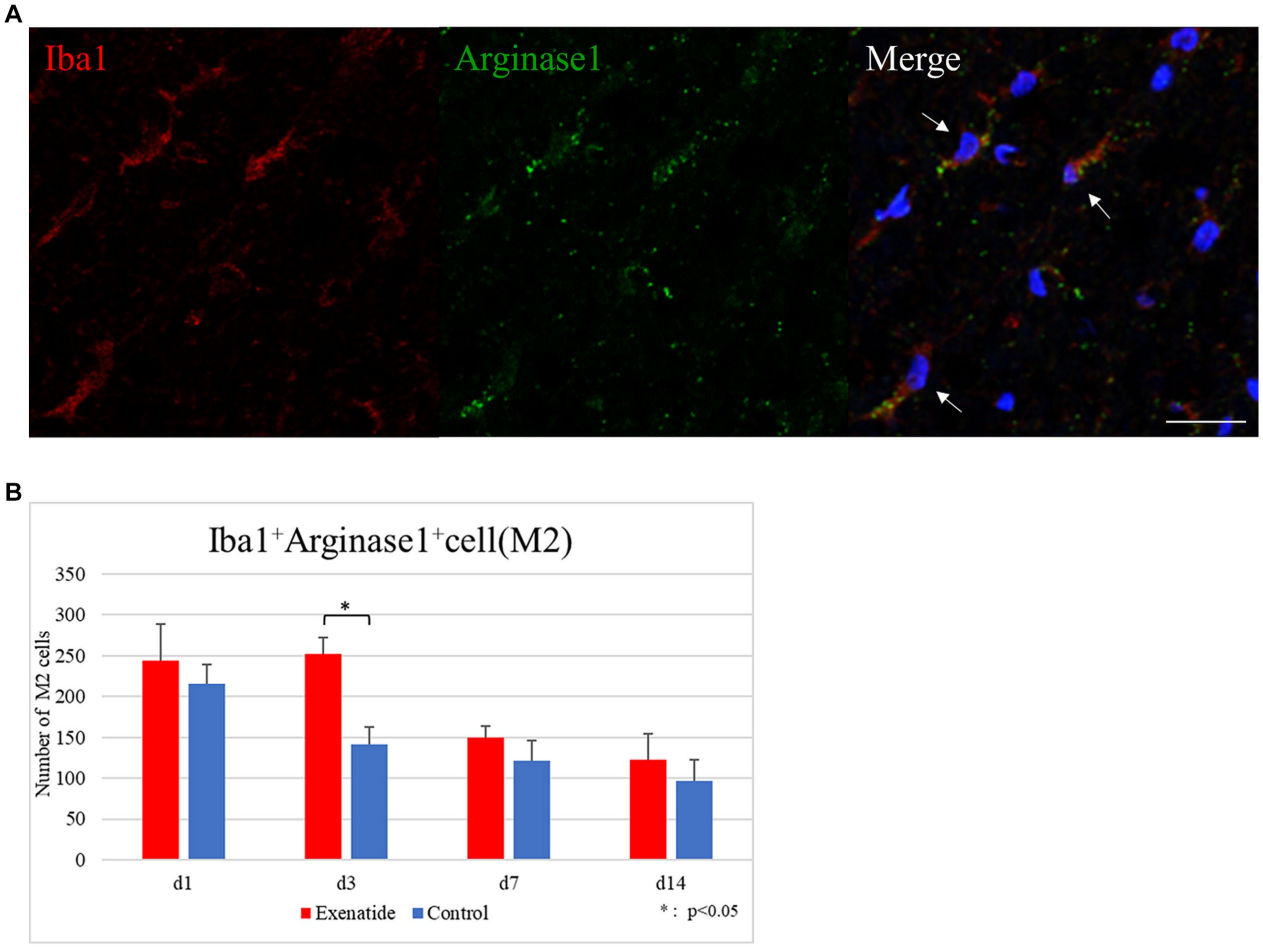
M2 macrophages in the injured spinal cord. **(A)** Representative immunohistochemical staining results for the M2 profile. DAPI: blue, Iba1: red, Arginase 1: green. Scale bar: 10 μm. **(B)** The number of Iba1+ Arginase 1+ cells in the posterior funiculus showing significantly higher numbers of M2 macrophages in the exenatide group on day 3 after injury. *n* = 5; error bar: SD; ^*^p < 0.05.

The number of infiltrating macrophages were quantified by immunohistochemistry. Quantification of macrophages withing the dorsal funiculus showed no significant difference in the number of Iba1+ iNOS+ M1 macrophage cells between the exenatide group and control group up to day 14 after injury (Figure 5A), but the number of Iba1+ Arginase 1+ M2 macrophage cells was significantly higher in the exenatide group on day 3 after injury (*p* < 0.05; Figure 6A).

## 4 Discussion

The pathophysiology of spinal cord injury is complicated. Following the primary injury which results from the physical trauma, a delayed but arguably more significant secondary injury process occurs within the injured spinal cord. The complex vascular, cellular, and biochemical processes that ensue during the secondary injury results in the apoptosis of neurons, oligodendrocytes, microglia, and astrocytes (Tator, 1998; Ramer et al., 2005; Oyinbo, 2011; Katoh et al., 2019). We previously reported that endoplasmic reticulum (ER) stress is an integral component of the secondary injury process and demonstrated that enhancement of the ER stress response improves recovery of motor function after SCI (Suyama et al., 2011; Kuroiwa et al., 2014; Matsuyama et al., 2014; Imai et al., 2018; Yanagisawa et al., 2019; Nomura et al., 2023). We first examined the benefits of enhancing the ER stress response by administering amiloride, a sodium-channel blocker that lowers blood pressure by acting as a potassium-retaining diuretic. Amiloride administration decreased the apoptosis of oligodendrocyte precursors cells, enhanced remyelination by oligodendrocytes that differentiated from surviving precursor cells, and improved motor function (Imai et al., 2018). Although the results were promising, the autonomic nerve dysfunction that is brought about by SCI impairs hemodynamic homeostasis, and a diuretic is not an ideal drug to be administered in an acute patient suffering from SCI. Having noted how GLP-1 receptor agonists enhanced the ER stress response in myocardial ischemia (Younce et al., 2013), kidney damage (Guo et al., 2017), cerebral infarction (Chien et al., 2015), and liver damage (Sharma et al., 2011), we decided to investigate the effects of exenatide, a GLP-1 receptor agonist capable of crossing the blood–brain barrier, in a rat model of SCI. We found that exenatide administration enhanced the ER stress response without adverse hypoglycemic effects, leading to a significant decrease in tissue damage and increased survival of oligodendrocyte precursor cells that brought about a significant improvement in motor function recovery. Hyperglycemia in the acute phase of SCI is reported to activate microglia that exacerbate the inflammatory response, induce apoptosis and demyelination, and impair improvement of motor function (Kobayakawa et al., 2014). While our previous study did not observe hyperglycemia in control animals (Nomura et al., 2023), hyperglycemia has often been observed in nondiabetic SCI patients (Kobayakawa et al., 2014), and GLP-1 receptor agonists may improve motor function by controlling hyperglycemia after SCI.

Although GLP-1 receptor agonists first came to prominence as a treatment for type-2 diabetes (Holst, 2004; Baggio and Drucker, 2007), similar protective effects have been confirmed on cells in the central nervous system, with reports describing the beneficial effects of GLP-1 receptor agonists in neurodegenerative diseases such as Alzheimer’s disease and Parkinson’s disease (Perry and Greig, 2002; Greig et al., 2004; Harkavyi et al., 2008; Aviles-Olmos et al., 2013; Ghasemi et al., 2013; Qiu et al., 2016; Kong et al., 2023; Koshatwar et al., 2023), the reduction of ischemic damage and maintenance of the blood–brain barrier after cerebral infarction (Li et al., 2009; Lee et al., 2011; Darsalia et al., 2014; Shan et al., 2019), and the recovery of motor function after SCI (Li H et al., 2015; Li Y et al., 2015; Li et al., 2016; Sun et al., 2018).

Many possible mechanisms for the neuroprotective effects of GLP-1 receptor agonists have been reported. In animal studies of stroke, exenatide has been shown to protect against ischemic stroke by upregulating GLP-1 receptors in neural cells (Lee et al., 2011), stimulating the ß-endorphin pathway (Jia et al., 2015), enhancing DNA repair efficiency (Yang et al., 2016), and promoting macrophage polarization toward the anti-inflammatory M2 phenotype among others (Kigerl et al., 2009; Darsalia et al., 2014). In animal studies of traumatic brain injury, exenatide and its analogs have been shown to attenuate hippocampal neuron loss (Heile et al., 2009), ameliorate H2O2-induced oxidative stress and glutamate toxicity (Rachmany et al., 2013), decrease Caspase-3 activity (Eakin et al., 2013), reduce reactive oxygen species (DellaValle et al., 2014), reduce pro-apoptotic signaling (Li H et al., 2015; Li Y et al., 2015), preserve the blood–brain barrier (Hakon et al., 2015), upregulate neurodegenerative disorder-related genes (Tweedie et al., 2016), maintain synaptophysin reactivity (Rachmany et al., 2017), and decrease glial activation and improve cognitive impairment (Bader et al., 2019). GLP-1 receptor signaling in astrocytes have been reported to improve mitochondrial integrity (Timper et al., 2020), reduce ER stress (Nomura et al., 2023), and reduce inflammatory cytokines produced by activated astrocytes (Iwai et al., 2006). Based on various reports noting that exenatide regulates inflammation through inflammatory cells such as macrophages and microglia (Kodera et al., 2011; Shiraishi et al., 2012; Darsalia et al., 2014; Wu et al., 2018; Lu et al., 2022), this study was performed under the premise that SCI is an inflammatory condition.

Although a wide variety of mechanisms take part in SCI secondary injury, inflammation is the most important factor implicated in the sequelae after SCI. Macrophages are central to the inflammation reaction, and their polarization is a topic that has garnered wide interest in the studies of SCI secondary injury. Macrophages accumulate within the hematoma of the injury epicenter immediately after SCI, but depending on their phenotype and activation status, they may exacerbate injury or promote repair. Classically activated M1 macrophages produce pro-inflammatory cytokines and cytotoxic mediators that increase their ability to kill pathogens within cells, while alternatively activated M2 macrophages produce large numbers of anti-inflammatory cytokines. Examining the polarization of macrophages in a mouse SCI model, Kigerl et al. reported that the biomarkers for both M1 and M2 macrophages increased after SCI with M1 macrophages being the majority during the early stage of SCI, but found that many biomarkers were transient and rapidly changed thereafter (Kigerl et al., 2009). Considering that treatments for SCI in the acute phase are limited, it is understandable that many trials are being conducted to modulate macrophage polarization towards benefitting the recovery process.

GLP-1 receptor agonists have been reported to affect macrophage polarization in a range of tissues, including the gastrointestinal tract (Werner, 2014), spinal cord (Qian et al., 2022), and peripheral blood (Buldak et al., 2016). Exenatide has also been reported to predominantly induce the production of M2 cells in models of cerebral infarction and neurodegenerative diseases such as Parkinson’s disease (Yu et al., 2023). This study was conducted to examine the effect of exenatide on macrophage polarization in the acute phase of SCI.

RT-PCR revealed that exenatide administration increased the expression of the M2 markers Arginase 1, CD163, and CD206 in the exenatide group on days 1 and 3 after injury (Figures 3A–C) and decreased the expression of the M1 markers iNOS and CD86 on day 3 and CD16 on day 1 after injury (Figures 2A–C), suggesting that exenatide shifts the polarization of macrophages infiltrating into the injured spinal cord to the anti-inflammatory M2 phenotype. This was accompanied by an increase in the expression of anti-inflammatory cytokines IL-4 and IL-10 on day 1 after injury (Figures 4C,D), which are implicated in the alternative activation of M2 macrophages (Mosser and Edwards, 2008; Chavez-Gala et al., 2015), and a decrease in the expression of inflammatory cytokines TNFα and IL-1β on days 1 and 3 after injury (Figures 4A,B), which are implicated in the classical activation of M1 macrophages (Chavez-Gala et al., 2015). The present study suggests that exenatide administration induces the production of M2 cells in the acute phase of SCI, and that motor function may be improved by the anti-inflammatory and tissue repair effects of these M2 macrophage cells. The fact that differences in macrophage markers as well as inflammatory and anti-inflammatory cytokines were only observed in the acute phase of the secondary injury (1–3 days post-injury) is most likely due to the fact that exenatide was only administered once immediately after injury, but also suggests that the beneficial effects of exenatide may be limited to the acute period following injury. Further studies employing multiple injections over the acute to subacute periods will be necessary to ascertain whether exenatide continues to provide anti-inflammatory or neuroprotective effects for a longer period.

Immunohistochemical staining was conducted to examine if this change was brought about mainly through a shift in the polarization or by a change in the numbers of macrophages with either polarity infiltrating into the injured spinal cord. Our results showed a significantly higher number of cells with the M2 expression profile in the exenatide group on day 3 after injury, but did not find a significant decrease in the number of cells with the M1 expression profile between the exenatide and control groups (Figures 5B, 6B). Taken at face value, this suggests that exenatide administration promotes the production or increases the infiltration of M2 phenotype macrophages, and that the increase of M2 cells with an anti-inflammatory/tissue repair effect is beneficial for the improvement of hindlimb motor function after SCI. However, the literature examining the effects of exenatide on macrophage polarity show that the promotion of microglial M2 polarization as seen in the increase of M2 markers does not necessarily coincide with a decrease in M1 markers (Darsalia et al., 2014; Wu et al., 2017). It is also possible that immunohistochemistry using another M1 antigen, for example CD86, may have shown a decrease because there have been reports in which exenatide significantly decrease CD86 but not iNOS (Darsalia et al., 2014).

This improvement in motor recovery brought about by one injection was also observed in our previous report (Nomura et al., 2023), and we feel that this illustrates the significance of targeting the secondary injury process of SCI as a point of intervention. For treatments aiming to mitigate the deleterious effects of the secondary SCI process, the earlier the treatment and more robust the effect, the greater the benefits to motor function recovery. Our previous study revealed that exenatide decreases endoplasmic reticulum stress and this study revealed that exenatide induces macrophage polarization to the cytoprotective M2 phenotype, decreasing the inflammatory response. However, we believe that the effects of exenatide administration after SCI are more wide-ranging, and we will continue to examine other facets of its effects.

There are several limitations of this study that need to be pointed out. One limitation is that we were unable to examine whether the observed effects were due to microglia endogenous to the spinal cord or monocytes derived from the blood and bone marrow. Furthermore, as all M2 cell subtypes were assessed together, the present study also could not clarify which M2 cell subtypes (from M2a through to M2d) were responsible for the observed effects. IL-4-activated M2a cells and IL-10-activated M2c cells are reported to have distinct phenotypes and functions (Mantovani et al., 2002), and further research evaluating the M2 cell subtypes is necessary. The effect of exenatide on transcription factors that are typically responsible for controlling M2 activation, such as signal transducer and activator of transcription 3 (STAT3), STAT6, interferon regulatory factor 4 (IRF4), and peroxisome proliferator activated receptor (PPAR) (Zhou et al., 2014; Chavez-Gala et al., 2015), is also a topic that requires future investigation. A second limitation of the study stems from the fact that these experiments were carried out with only female rats. This is a conscious choice based upon a long-standing belief that female rodents suffer less from bladder infection while their bladder function is impaired from spinal cord injury. We are aware of studies that have reported on gender differences in the recovery process from SCI (Datto et al., 2015), and so raise the possibility of gender bias as a limitation of this study. A third limitation concerns the immediate administration of exenatide after SCI as well as the dosage. While the results of this study demonstrate the beneficial effects of exenatide administration immediately after SCI, this is not feasible for human patients and the administered dose is significantly higher than the current clinical dosage for diabetes. Therefore, further studies will be necessary to determine the therapeutic time window of exenatide administration following SCI as well as its effect at clinical dosage. A fourth limitation stems from the fact that only changes in macrophages were investigated. Since GLP-1 receptors have been broadly found across most types of neuronal cells within the brain, spinal cord, and ganglion and peripheral nerve (Perry et al., 2007; Li et al., 2012; Salcedo et al., 2012), further studies will be necessary to glean the full scope of the effects brought about by exenatide administration.

Significant advances in post-trauma management and rehabilitation have improved the general prognosis of SCI, but the functional improvements brought about by current treatment options are unfortunately still limited. While cell replacement therapy utilizing engineered stem cells is anticipated to become a viable therapy in the near future, significant technical, financial, and geographical constraints are expected to limit stem cell treatments to a select few large clinical research facilities, denying access to many patients suffering from the debilitating effects of SCI. Furthermore, it seems more logical to try to prevent the loss of neurological tissue than to try to reconstruct that which we cannot create in the first place. This has been the impetus for developing treatments that protect the traumatically injured brain or spinal cord from the extremely destructive secondary injury process and to save as much neural function as possible. It is unfortunate that all of our collective research has not yielded on option to replace the decades-old corticosteroid protocol that is now mostly abandoned. Our group has been researching this field originally with a focus on increasing the endoplasmic reticulum stress response to enhance the cellular homeostasis mechanisms and provide the injured spinal cord with an increased capacity to weather the secondary injury storm. We stumbled upon GLP-1 receptor agonists as an approved diabetes drug that had been shown to decrease endoplasmic reticulum stress within the pancreatic islet cells and other organs. We did not expect that GLP-1 receptor agonists would turn out to have the numerous beneficial effects on degenerative or traumatic neurological conditions, or that it would garner such wide-spread societal interest as a weight-loss agent. The findings of this study along with the numerous studies that have been published in the field of traumatic brain injury raises hope that GLP-1 receptor agonists may very well be the drug that we have been searching for. While hopeful, there remains much to be studied before GLP-1 receptor agonists may be clinically used to treat SCI patients. For example, we do not know the therapeutic time window or the optimal timing, dosage, or duration of GLP-1 receptor agonist administration that would bring about maximal effect in rodents or how to translate that into human trials. While the path forward may be treacherous, we hope that our efforts will play some part to advance the treatment options for patients that unfortunately suffer a spinal cord injury.

## Data availability statement

The original contributions presented in the study are included in the article/supplementary materials, further inquiries can be directed to the corresponding author.

## Ethics statement

The animal study was approved by Tokai University Teaching and Research Support Center. The study was conducted in accordance with the local legislation and institutional requirements.

## Author contributions

TN: Writing – original draft, Conceptualization, Data curation, Investigation, Methodology. HK: Writing – review & editing, Data curation, Funding acquisition, Methodology, Project administration, Supervision, Validation, Visualization. SN: Writing – review & editing, Conceptualization, Data curation, Methodology, Writing – original draft. KO: Writing – review & editing, Validation, Visualization. MW: Supervision, Writing – review & editing, Funding acquisition.
